# Supplementary material for: Telemedicine and AI-Powered Chatbots: Potential and Challenges for Home Care Provided by Family Caregivers
Source: Healthcare (Basel). 2025 Dec 3;13(23):3159. doi: 10.3390/healthcare13233159 (PMC12692227; doi:10.3390/healthcare13233159)
Supplement: Supplementary file 1 [file healthcare-13-03159-s001.zip › healthcare-3972687-supplementary.pdf]

Additional materials

Table S1. List of all items in Study 1.

Item	Question	i-CVI
Q1	Do you already have experience with using telemedicine?	0.88
Q2	Did you find it helpful in caring for people in need of care?	0.87
Q3	What was your overall experience with using telemedicine?	0.89
Q4	What condition does the person in need of care whom you care for have?	0.90
Q5	How important would stable internet access be to you for using telemedicine?	0.91
Q6	Do you have a suitable device (computer, tablet, or smartphone) for using it?	0.88
Q7	How important would intuitive and easy-to-understand operation of telemedicine be to you?	0.87
Q8	How likely would you be to use telemedicine if technical support were available?	0.89
Q9	To what extent would technical difficulties affect your willingness to use telemedicine?	0.88
Q10	Would success stories and positive experiences from other users increase your willingness to use it?	0.87
Q11	What technical barriers could prevent you from using telemedicine?	0.89
Q12	What fears or concerns do you have about using telemedicine?	0.88
Q13	How could technical support be designed to make you feel safe?	0.87
Q14	What role does support from family, friends, or neighbors play in the introduction of new technologies?	0.89
Q15	What would need to be done to increase your confidence in using telemedicine?	0.88
Q16	How important would the protection of your personal and medical data be to you when using telemedicine?	0.87
Q17	How much would reports of data leaks or cyberattacks influence your decision?	0.89
Q18	How helpful would it be to you if all data transmitted via telemedicine were encrypted?	0.88

Q19	Would you be more likely to use telemedicine if the technologies used were certified according to recognized security standards?	0.87
Q20	How much would incomprehensible or complex privacy policies affect your willingness to use telemedicine?	0.89
Q21	How important would the introduction of two-factor authentication be to you in order to secure your access to telemedicine systems?	0.88
Q22	What specific concerns do you have about data protection when using telemedicine?	0.87
Q23	What measures could help you gain confidence in the data security of telemedicine systems?	0.89
Q24	How could data protection policies be designed to make them more understandable and transparent for you?	0.88
Q25	What kind of information or training on data protection would you need to feel confident enough to use telemedicine?	0.87
Q26	To what extent would it be important for you to be notified in real time about possible data protection or security incidents?	0.89
Q27	How important would regular training on the use of telemedicine technologies in home care be to you?	0.88
Q28	How helpful would it be for you if a technical hotline were available around the clock?	0.87
Q29	How likely would you be to use telemedicine if regular workshops and training courses were offered?	0.89
Q30	How much would technical difficulties affect your motivation to use telemedicine?	0.88
Q31	How important would a user-friendly and easy-to-understand interface be to you when using telemedicine systems?	0.87
Q32	Would visual aids such as online tutorials or manuals help you to better understand and use telemedicine technologies?	0.89
Q33	What kind of training would you need to feel confident enough to use telemedicine technologies?	0.88
Q34	What challenges or difficulties do you expect when using telemedicine in home care?	0.87
Q35	How could technical support be designed to best help you solve problems quickly?	0.89
Q36	What improvements would need to be made to the design of telemedicine applications to make them easier for you to use?	0.88

Q37	How important would it be for you to exchange ideas with other family caregivers in order to benefit from their experiences with telemedicine?	0.87
Q38	How important would regular and clear communication with the medical team regarding the health status of the person in need of care be to you?	0.89
Q39	How helpful would a special telemedicine platform be that enables direct and secure communication and information exchange with the medical team?	0.88
Q40	How likely would you be to use telemedicine if it guaranteed direct and timely communication with the medical team?	0.87
Q41	How often have you had difficulty receiving timely feedback from the medical team in the past?	0.89
Q42	How important would an emergency communication plan be to you, enabling you to contact the medical team immediately in critical situations?	0.88
Q43	What difficulties have you experienced in the past when communicating with the medical team?	0.87
Q44	How could communication with the medical team be improved to avoid misunderstandings and delays?	0.89
Q45	What features should a communication platform offer to enable you to interact securely and efficiently with the medical team?	0.88
Q46	How important would it be for you for the medical team to provide regular feedback on the person requiring care, and how often do you think this feedback should be provided?	0.87
Q47	What do you expect from the integration of a telemedicine communication platform in terms of the quality and speed of communication?	0.89
Q48	How old are you?	0.88
Q49	What is your gender?	0.87
Q50	What is your highest level of education?	0.89
Q51	How long have you been caring for a loved one?	0.88
Q52	How many hours per week do you spend on average providing care?	0.87

Table S2. List of all items in Study 2.

Item	Question	i-CVI
Q1	How helpful would an AI-powered chatbot be for technical support when using telemedicine?	0.92
Q2	How helpful would an AI-powered chatbot be for emotional support when using telemedicine?	0.91
Q3	How helpful would an AI-powered chatbot be for providing general information about telemedicine?	0.90
Q4	How important would the user-friendliness of an AI-powered chatbot be to you?	0.93
Q5	How important would the availability of an AI-powered chatbot around the clock be to you?	0.92
Q6	How much do you trust the reliability of an AI-powered chatbot?	0.91
Q7	How much do you trust the security of an AI-powered chatbot?	0.90
Q8	How helpful would it be for you if an AI-powered chatbot could provide stress management tips?	0.92
Q9	How helpful would it be for you if an AI-powered chatbot could share success stories from other users?	0.91
Q10	How important is the protection of your personal and medical data when using an AI-powered chatbot?	0.90
Q11	How much would concerns about data protection affect your willingness to use an AI-powered chatbot?	0.93
Q12	How helpful would it be for you if all data transmitted via the chatbot were encrypted?	0.92
Q13	How important would the introduction of two-factor authentication be to you to secure your access to an AI-powered chatbot?	0.91
Q14	How much would incomprehensible or complex privacy policies affect your willingness to use an AI-powered chatbot?	0.90
Q15	What measures could help you gain confidence in the data security of an AI-powered chatbot?	0.93
